# Challenges in diagnosis and treatment: a case report on a mixed malignant Müllerian tumor

**DOI:** 10.1093/jscr/rjad523

**Published:** 2023-09-16

**Authors:** Daniela Saenz, Jorge D Fernández, Juan P Cóbar

**Affiliations:** Department of Medical Research, Universidad Francisco Marroquín, Guatemala City 01010, Guatemala; Department of Medical Research, Universidad Francisco Marroquín, Guatemala City 01010, Guatemala; Department of Medical Research, Universidad Francisco Marroquín, Guatemala City 01010, Guatemala

**Keywords:** Müllerian tumor, gynecologic malignancy, abnormal uterine bleeding

## Abstract

Mixed malignant Müllerian tumors (MMMTs) are rare and aggressive neoplasms made up of both carcinomatous and sarcomatous components that primarily appear in the female reproductive tract. The cellular origin of this malignancy has eluded advancements in molecular and immunohistochemical techniques contributing to the limited diagnostic and therapeutic strategies. This case report presents a 41-year-old female with a history of abnormal uterine bleeding and dysmenorrhea who was later diagnosed with an MMMT. This case highlights the importance of considering MMMTs in patients with a long-standing history of abnormal uterine bleeding because the prompt recognition and diagnosis of this condition may lead to an improved overall survival for these patients.

## Introduction

Mixed malignant Müllerian tumors (MMMTs) are rare and aggressive neoplasms that arise in the female reproductive tract [1]. They represent a complex interplay of carcinoma and sarcoma components, demonstrating both epithelial and mesenchymal differentiation. MMMTs are most commonly encountered in the uterus but have also been reported in other locations such as the ovaries, fallopian tubes, and peritoneum [2]. They account for a small fraction of gynecological malignancies; MMMTs pose a significant clinical challenge due to their aggressive behavior, propensity for distant metastasis, and limited treatment options.

Historically, MMMTs have been subject to a variety of terminologies, including carcinosarcoma, malignant mixed Müllerian tumor, and metaplastic carcinoma [3, 4]. This ambiguity in nomenclature further reflects the complexity surrounding the classification and understanding of these tumors. Despite advancements in molecular and immunohistochemical techniques, the exact cellular origin and oncogenesis of MMMTs remain elusive, proving difficult to establish targeted therapies and prognostic factors [3].

The following case report presents a 41-year-old woman diagnosed with MMMT, providing a detailed description of the patient’s presentation, workup, and management.

## Case report

A 41-year-old woman presented with a 5-day history of abnormal vaginal bleeding. She reported that, 10 months ago, she developed metrorrhagia associated with dysmenorrhea. Her past medical history revealed uterine fibroids, previously treated with myomectomy. Physical examination revealed a normal cervix and vaginal walls, moderate transcervical hemorrhage through a closed cervical os, retroverted uterus, and normal adnexa. An ultrasound revealed a retroverted uterus of 11.3 × 8.2 × 7 cm, heterogeneous endometrium of 23.3 mm, normal ovarian dimensions, and no free fluid. Subsequently, cervicovaginal cytology studies were performed, showing moderate inflammatory changes, but these were negative for intraepithelial lesions or malignancy. Since cervical changes were not evident, an endometrial biopsy was performed, which diagnosed endometrial hyperplasia and revealed a malignant tumor of the body of the uterus.

A computed tomography of the abdomen and pelvis revealed no evidence of metastasis, and an exploratory laparotomy was performed together with a total abdominal hysterectomy, salpingectomy, omentectomy, pelvic node dissection, and peritoneal biopsy.

**Figure 1:**
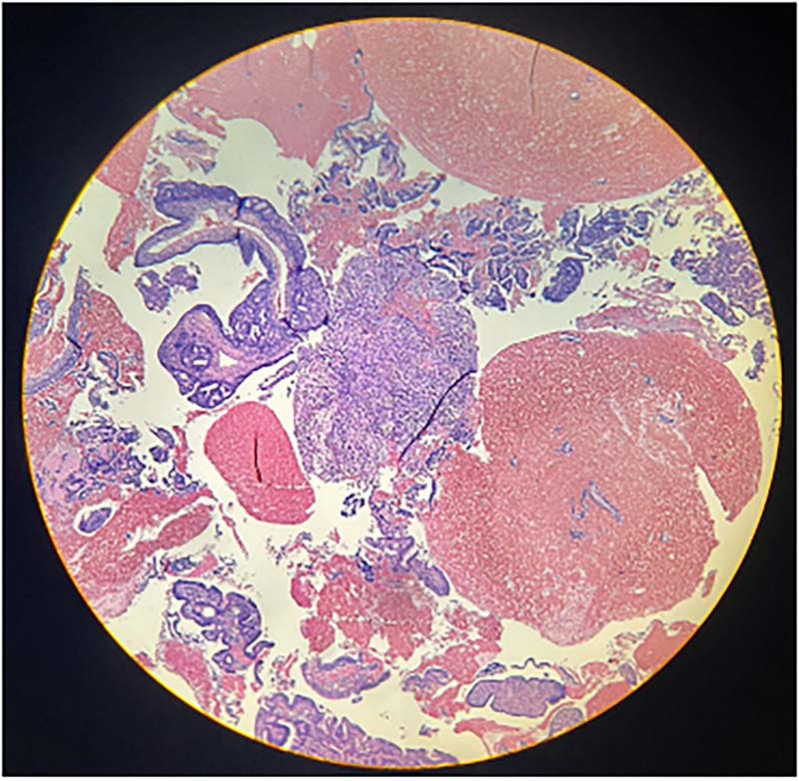
Photomicrograph shows the presence of a malignant mixed tumor containing epithelial and sarcomatous components (H&E stain ×4).

**Figure 2:**
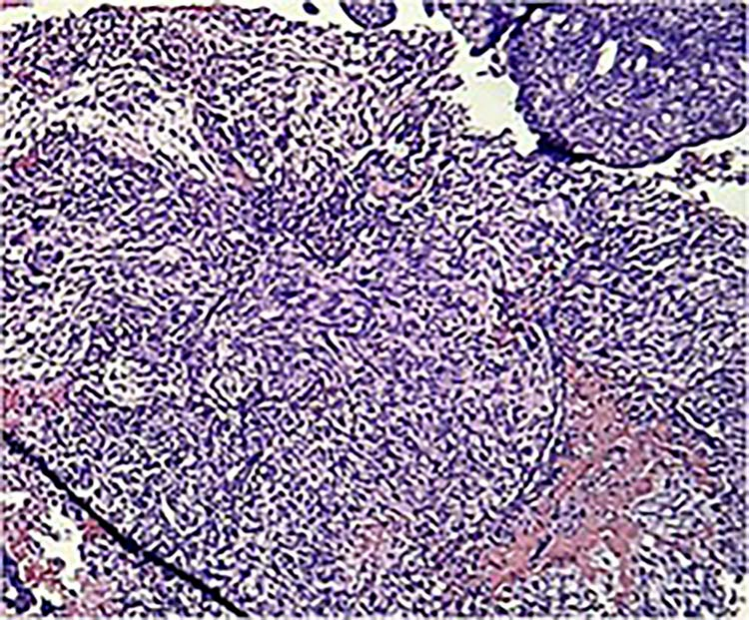
Photomicrograph shows homologous sarcomatous components, composed of sarcomatous spindle shaped cells, with elongated nuclei and irregular nuclear membrane. (H&E stain ×10).

**Figure 3:**
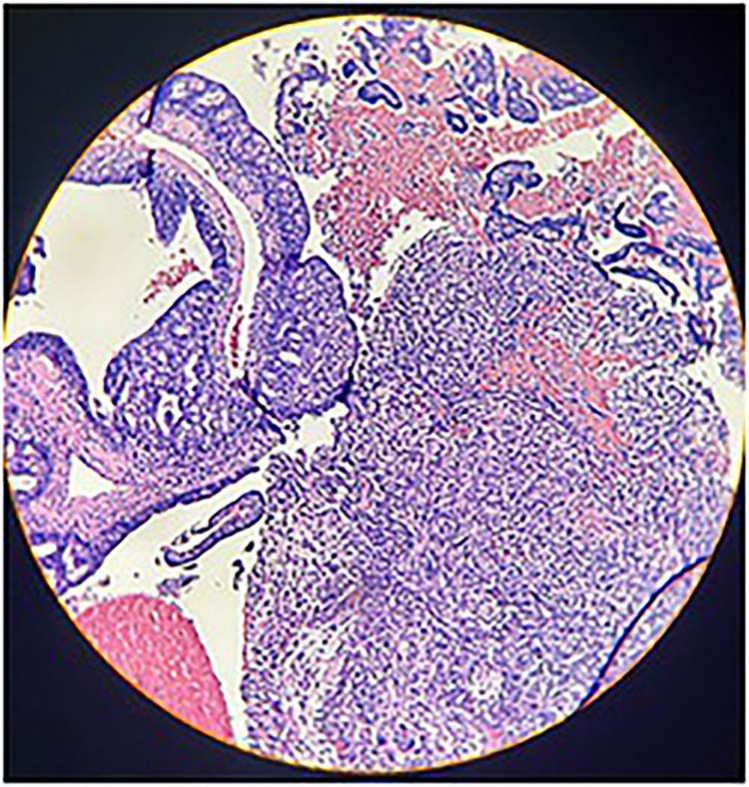
Photomicrograph shows epithelial components that form atypical glands and solid nodules with a sarcomatous component (H & E stain ×10).

Macroscopically, an amorphous uterus weighing 397 g and measuring 12 × 9 × 7.5 cm with a brown serosa, and multinodular violaceous areas was examined. When incised, the endometrial cavity was occupied by a whitish polypoid mass of 8 × 5 cm which invaded the myometrium. In addition, multiple firm whitish nodules ~2 × 1.5 cm in diameter were identified. Bilateral ovaries and fallopian tubes appeared to be of normal morphology and size. Histologically, there was evidence of epithelial and sarcomatous components (Figs 1 and 2), and the epithelial component developed atypical glands (Fig. 3). The diagnosis of MMMT was given due to histologic findings.

## Discussion

Uterine carcinosarcoma was considered as a type of uterine sarcoma and is called as malignant mixed Müllerian tumor or mixed mesodermal sarcoma; however, these neoplasms are now classified as carcinomas because they derive from monoclonal neoplastic cells [5]. The risk factors for MMMTs are similar to those of endometrial carcinomas and include obesity, nulliparity, and use of exogenous estrogens and tamoxifen. The use of progestogen-containing contraceptives protects against both types of neoplasms [1, 6].

The diagnosis of MMMT is achieved through histological evaluation, although clinically, patients may present with a classic clinical triad of pain, bleeding, and a rapidly enlarging uterus. Vaginal bleeding is the most common finding, although not specific [1, 4]. In this case, the patient presented with a 5-day history of abundant vaginal bleeding, dating back to 10 months of abnormal uterine bleeding and dysmenorrhea.

On microscopic examination, it was characterized by the coexistence of malignant epithelial and mesenchymal components, which posed significant challenges for accurate identification and classification [7]. MMMTs are regarded for their molecular and genetic heterogeneity, which contributes to the variability in clinical behavior and treatment responsiveness [8]. The lack of specific biomarkers for MMMTs limits our accuracy for diagnosis and personalization of treatment strategies [5, 8].

There are many hypotheses on the tumor’s pathogenesis. The “collision theory” states that the tumor originates from two distinct malignant cellular populations; the “combination theory” proposes a common cell with the ability to differentiate along both lines; and the “metaplastic monoclonal or conversion theory” describes a metaplastic transformation of a single cell type [2]. In a study that performed genomic, epigenomic, transcriptomic, and proteomic characterizations of MMTs, frequent mutations were found in TP53, PTEN, PIK3CA, PPP2R1A, FBXW7, and KRAS, such as with endometrioid and serous uterine carcinomas [9].

The primary management of MMTs limited to the abdomen is surgery for staging and initial treatment, including total hysterectomy, bilateral salpingo-oophorectomy, and pelvic and para-aortic lymph node dissection. Lymphadenectomy should be done in all patients with carcinosarcoma not only for staging purposes but because of the associated improvement in overall survival [1].

Uterine carcinosarcoma is surgically staged according to the 2017 International Federation of Gynecology and Obstetrics/Tumor, Node, Metastasis classification system. The approach to adjuvant treatment following surgical resection is based on the stage of diagnosis:

- Stage IA: Adjuvant therapy is uncertain; however, some experts prefer to keep patients under observation.

- Stages IB–IV: Supporting evidence is limited, but experts suggest combination platinum-based chemotherapy. Carboplatin and paclitaxel are recommended for patients with recently diagnosed uterine carcinosarcoma who have undergone surgical removal.

In the advanced-stage disease, it is reasonable to monitor CA-125 every 3 months and compare it with preoperative levels [9]. The 5-year survival rate for Stage III carcinosarcomas is ~30% and only 50% is diagnosed at Stage I [1].

## Conclusion

MMMTs are infrequent and aggressive tumors that primarily occur in the uterus. They are known as carcinosarcomas due to their unique histological composition of both malignant epithelial and mesenchymal components. Most cases present with abnormal vaginal bleeding and pain, as evidenced in our case report. Various imaging strategies may be employed for prompt diagnosis, but endometrial biopsy with a histopathological examination remains the gold standard.

This case report underscores the complexity and diagnostic challenges associated with this infrequent malignancy. The histopathological and molecular features of MMMTs require further investigation and multidisciplinary collaboration for the development of diagnostic and therapeutic approaches.

## Author contributions

All authors meet authorship guidelines based on the following contributions: D.S., J.D.F., and J.P.C.: manuscript prep and revision and is accountable for all aspects of work; J.P.C.: final approval.

## Conflict of interest statement

This statement affirms that the authors have no financial, personal, or other interests that could influence the research or its interpretation.

## Funding

None declared.

## Data availability

This statement indicates that the data related to the case report can be made available to interested parties upon request to ensure transparency and facilitate further examination if necessary.

## Informed consent

An informed consent form was signed prior to the inception of this case report.
